# Gastrointestinally Digested Protein from the Insect *Alphitobius diaperinus* Stimulates a Different Intestinal Secretome than Beef or Almond, Producing a Differential Response in Food Intake in Rats

**DOI:** 10.3390/nu12082366

**Published:** 2020-08-07

**Authors:** Alba Miguéns-Gómez, Carme Grau-Bové, Marta Sierra-Cruz, Rosa Jorba-Martín, Aleidis Caro, Esther Rodríguez-Gallego, Raúl Beltrán-Debón, M Teresa Blay, Ximena Terra, Anna Ardévol, Montserrat Pinent

**Affiliations:** 1 MoBioFood Research Group, Departament de Bioquímica i Biotecnologia, Universitat Rovira i Virgili, c/Marcel·lí Domingo n 1, 43007 Tarragona, Spain; alba.miguens@urv.cat (A.M.-G.); carme.grau@urv.cat (C.G.-B.); marta.sierra@urv.cat (M.S.-C.); esther.rodriguez@urv.cat (E.R.-G.); raul.beltran@urv.cat (R.B.-D.); mteresa.blay@urv.cat (M.T.B.); ximena.terra@urv.cat (X.T.); montserrat.pinent@urv.cat (M.P.); 2 Servei de Cirurgia General i de l’Aparell Digestiu, Hospital Universitari Joan XXIII, 43007 Tarragona, Spain; rosa.jorba1@gmail.com (R.J.-M.); dra5028@gmail.com (A.C.); 3 Institut d’Investigació Sanitària Pere Virgili (IISPV), 43007 Tarragona, Spain

**Keywords:** dietary protein, in vitro digestion, gut, enterohormones, food intake, insect, almond, beef

## Abstract

In this study we compare the interaction of three protein sources—insect, beef, and almond—with the gastrointestinal tract. We measured the enterohormone secretion ex vivo in human and pig intestine treated with in vitro digestions of these foods. Insect and beef were the most effective in inducing the secretion of CCK, while almond was the most effective in inducing PYY in pig duodenum. In the human colon, almond was also the most effective in inducing PYY, and GLP-1 levels were increased by insect and beef. The three digested proteins reduced ghrelin secretion in pig duodenum, while only insect reduced ghrelin secretion in human colon. We also found that food intake in rats increased in groups fed a raw insect pre-load and decreased when fed raw almond. In conclusion, the insect *Alphitobius diaperinus* modulates duodenal and colonic enterohormone release and increases food intake in rats. These effects differ from beef and almond.

## 1. Introduction

The world population is predicted to reach 9.7 billion inhabitants in 2050 [1]. This presents a major challenge that can be faced through more environment-friendly food and feed production models. In the twenty-first century, insects as food and feed are emerging as an alternative solution to conventional livestock. The mass production of insects is economically viable and presents some environmental advantages, such as the high feed conversion efficiency, emission of fewer greenhouse gases and less ammonia, and they require less land and water than cattle rearing [2,3]. As food or feed, insect protein could complement animal protein to address the challenge of feeding a rising population [4,5].

Consuming the recommended quantity of good-quality protein is essential for optimal human growth, development, and health [6]. Some research has characterized insects as an abundant source of high-quality protein and essential amino acids [7]. Although entomophagy has been a long-standing part of life in many cultures [2], other societies still reject the practice primarily due to cultural biases towards consuming insects and a lack of knowledge about this novel food [2]. The nutritional value of insects has been extensively documented [8,9]. In addition to protein quality and quantity, they have high-quality fat content with high MUFA and/or PUFA content, and are rich in minerals and vitamins [10,11,12]. Although this composition varies depending on the insect’s species [9], sex [13], and diet [14], edible insects are considered a nutritious alternative to traditional animal protein derived from livestock. However, there is a lack of information about their bioactive properties. Some studies, mostly *in vitro*, have examined the effects of edible insects on human health, including their interaction with microbiota [15], antihypertensive peptides [16], and their antimicrobial function [17], among other health benefits [18,19]. Nevertheless, the possible effects of insect intake at the gastrointestinal level have rarely been the focus of study.

The gut is the largest endocrine organ in the body [20], and secreted enterohormones are involved in a wide range of physiological and metabolic processes, such as appetite regulation, gastric motility, and glucose homeostasis. The enteroendocrine cells (EECs) present in the gastrointestinal tract interact with nutrients and secrete hormones in response to food ingestion, acting as chemosensors of the lumen content [21]. EECs are scattered throughout the gastrointestinal tract, and the hormones they produce are concentrated in specific regions of the gut, according to their roles in regulating these physiological functions [22,23]. Each gut hormone appears in the bloodstream based on its individual temporal profile, which is determined by patterns of food intake, nutrient absorption, and the distribution of EECs along the intestinal tract [24,25]. Dietary compounds can modulate enterohormone secretion. For example, protein hydrolysates, or even intact protein, induce the secretion of satiety hormones. In the body of literature on proteins, dairy protein is one of the most studied: casein and whey proteins have been shown to increase GLP-1, CCK, and PYY plasma levels in human subjects [26,27]. Other plant and animal protein sources have also demonstrated their capacity to enhance gut hormone secretion in in vitro and in vivo experimental conditions [28,29,30].

In this study, we aimed to compare how animal proteins from insects and beef and plant protein from almonds interact with the gastrointestinal tract. We focused our analysis on how they act on the enteroendocrine system and food intake regulation. We assayed their effects on the secretome in ex vivo models of human and pig intestine treated with in vitro digestions of these protein sources. We next assayed the satiety capacity of raw insect, beef, and almond in vivo in rats.

## 2. Materials and Methods

### 2.1. Chemicals and Reagents

Raw insect (Buffalo *Alphitobius diaperinus* powder) and IPC (*Alphitobius diaperinus* insect protein concentrate powder) were provided by Protifarm. Beef (a lean portion, Protifarm NV, Ermelo, the Netherlands) was purchased at a local market (Mercat Central, Tarragona, Spain) and almond (*Prunus dulcis*) flour was provided by Borges Agricultural & Industrial Nuts (BAIN). The nutritional composition of these samples, provided by producers or bibliography [31], is presented in Table S1. All the samples were stored in the dark at −20 °C for optimal conservation.

Chemicals, porcine digestive enzymes (α-amylase, pepsin, and pancreatin, Sigma-Aldrich, Madrid, Spain) together with bile salts were purchased from Sigma-Aldrich (Madrid, Spain). Pancreatin contains enzymatic components including trypsin, amylase, lipase, ribonuclease, and protease.

The ELISA kits for acyl-ghrelin (catalog no. EZRGRA-90K), total ghrelin (catalog no. EZGRT-89K), and total GLP-1 (catalog no. EZGLPT1-36K) were purchased from Millipore (Billerica, MA, USA). ELISA kits for CCK (catalog no. EKE-069-04), PYY (catalog no. FEK-059-03), and human PYY (catalog no. FEK-059-02) were purchased from Phoenix Pharmaceuticals (Burlingame, CA, USA). We used them according to the instructions provided by producers that could be easily obtained in the websites of each enterprise with detailed references (see Table S2). We measured all the samples in the sample plate for each hormone (intra-assay variation detailed in Table S2).

### 2.2. In Vitro Digestion

Foods were digested according to the INFOGEST harmonized protocol [32], first published in 2014 by Minekus et al. [33], which consists of the simulation of the three main stages of in vivo digestion: the oral, gastric, and intestinal stages. In our study, we used samples digested up to the gastric phase (gastric digestion) and totally digested samples (intestinal digestion). The two types of digestion occurred in parallel. Food quantity was adjusted by protein content in order to achieve the same ratio of protein per volume for all the samples (0.12 g protein/mL simulated saliva).

#### 2.2.1. Gastric Digestion

The different foods were minced with 20 mL of simulated saliva using a mincer (Ultra-Turrax T25; IKA Werke, Staufen, Germany) for 2–5 min. Amylase was added after this step (75 U mL^−1^) and mixed with the minced food for 2 min. Then, 20 mL of simulated gastric juice containing pepsin (2000 U mL^−1^) was added and mixed for 120 min. Half of the gastric digestion samples were stopped at this point, and the other half went on to the next step.

#### 2.2.2. Intestinal Digestion

To 20 mL of gastric digestion sample, 20 mL of simulated intestinal juice containing pancreatin (100 U mL^−1^) and bile salts (10 mM) was added and incubated for 120 min.

We also applied the same procedures to all four food samples but without enzymes as a negative control for digestion, and as an enzyme control we followed the procedure using only the enzymes and simulated fluids without food. Finally, we placed all the digestions and the controls in a 90 °C bath for 20 min to stop the enzymatic reactions. After that we minced and centrifuged all the samples (4000 rpm, 5 min, 4 °C) to discard the undigested fractions: a pellet for all samples and an upper layer of fat for the almond and insect digestions. The samples were stored at −20 °C.

### 2.3. Characterization of Digestion Products

The glucose and triglyceride contents of the digested samples, negative controls, and enzyme controls were quantified using commercial kits obtained from QCA (Amposta, Spain). A BCA kit was used (Pierce, Thermo Fisher Scientific) to measure protein content.

SDS-PAGE electrophoresis was performed to test the digestion process. Thirty microliters of digested sample (protein concentration of 2 μg/μL) was mixed with 10 microliters of 4 × sample loading buffer (125 mM Tris HCl (pH 6.8), 2.5% (*w/v*) sodium dodecyl sulfate (SDS), 0.1% (*w/v*) bromophenol blue, 25% (*v/v*) glycerol, 25% (*v/v*) β-mercaptoethanol) and heated at 100 °C for 5 min. From this, fifteen microliters were loaded on a 16% polyacrylamide gel. A molecular weight marker (Page Ruler, Thermo Fisher Scientific) and undigested samples were included on each gel. Gels were then stained with colloidal Coomassie Blue (Bio-Rad Laboratories).

### 2.4. Tissue Collection

#### 2.4.1. Pig Tissue

Intestinal segments of duodenum were obtained from three female pigs (*Sus scrofa domesticus, Landrace X Largewhite)* that were slaughtered for meat production at a local slaughterhouse. The pigs were commercial breeds that weighed approximately 120 kg at slaughter and had been fasted for approximately 24 h prior to slaughter. The procedure for collecting and preparing the tissue for the experiment was previously established by Ginés et al. (2018) [34]. Five minutes after slaughter, the intestines were excised and segments of the anatomical regions required for the experiment (duodenum) were placed in ice-cold oxygenated (95% O_2_, 5% CO_2_) Krebs–Ringer bicarbonate (KRB) buffer (pH 7.4) with D-mannitol 10 mM (Sigma Aldrich, Madrid, Spain). The tissues were promptly transported to the laboratory at 4 °C and immediately used for the experiments. The time elapsed between excision and the beginning of the experiments was about 30 min.

#### 2.4.2. Human Tissue

Human colon segments were collected from 10 donor patients of both sexes (median age 65 years) who had undergone colon surgery due to colorectal carcinoma. Only subjects who satisfied the study criteria were selected. The exclusion criteria were the consumption of anti-inflammatory drugs, alcohol abuse, and intestinal bowel disease or celiac disease, as these would alter intestinal functioning. The characteristics of the patients included in this study are summarized in Table S3 of the supplementary material. All donor patients provided informed consent and the study was approved by the Clinical Research Ethics Committee (CEIC) of the Hospital Universitari Joan XXIII in Tarragona (CEIm 101/2017). Healthy tissues not needed for diagnostic purposes were excised from the proximal and distal colon. After resection, these colon tissues were transferred from the hospital within 30 min in ice-cold oxygenated KRB buffer (pH 7.4) with D-mannitol 10 mM. This tissue collection protocol was previously established by González-Quilen et al. (2020) [35].

### 2.5. Ex Vivo Experiments with Intestinal Segments

After rinsing the pig duodenum with KRB buffer, it was mounted on a plastic tube to facilitate stripping—the removal of the serosal and outer muscular layers—with a scalpel. The intestinal tube was then sliced longitudinally and circles of tissue with a diameter of 14 mm were taken using a biopsy punch. This process took approximately 15 min. The sample was kept at a low temperature with cold buffer and an ice-cold bath during the entire procedure. We then started the secretion study. We placed each duodenum circle in a well (24-well plate) containing 1 mL of KRB buffer with D-mannitol 10 mM pre-warmed to 37 °C for 15 min. After this pre-incubation period, the buffer was replaced by the same volume of pre-warmed treatments, diluted in KRB buffer containing glucose 10 mM (Sigma Aldrich, Madrid, Spain), protease inhibitors aprotinin 100 KIU (Sigma Aldrich, Madrid, Spain) and amastatin 10 μM (Enzo Life Sciences, Madrid, Spain), and 0.1% BSA (Sigma Aldrich, Madrid, Spain). The control group was treated with the same solution (KRB buffer with glucose) without any additions. The incubation period was 60 min for the PYY and CCK secretion studies and 90 min for the active ghrelin secretion study. In this case, as we were studying the enterohormonal secretion profile in pig duodenum, we used the gastric digestions as treatments. We simultaneously assayed all the treatments in the same animal tissue. These tissue explants were divided into five treatment groups: control (*n* = 3 replicates), enzyme control, insect, beef, and almond (*n* = 2 replicates). For all the digested samples, the protein content was adjusted to a dose of 15 mg/mL, as we have previously reported its effectiveness in modulating enterohormone secretion in intestinal segments [36]. The enzyme control was diluted by half, using the same dilution factor as for the digested insect sample to have a similar enzyme concentration as in treatment groups. After the incubation period, the medium was collected and stored at −80 °C in different aliquots for further analysis.

The tissue processing and the experimental protocol used for the human colon was largely the same as described for the pig tissue. The colon explants, measuring 5 mm in diameter, were placed in a 48-well plate, previously filled with 400 μL KRB buffer with D-mannitol. In this experiment, we used the intestinal digestions diluted to 5 mg/mL protein content. For each human donor sample, we obtained six colon explants that were divided into six groups: control, enzyme control, insect, IPC, beef, and almond. The enzyme control was diluted by one-third as in the digested insect sample. After a 30 min incubation period, the medium was collected and stored at −80 °C in different aliquots.

### 2.6. Food Intake Study in Rats

Ten female RccHan:WIST rats (8 weeks old, 220–240 g) were obtained from provider ENVIGO (Castellar del Vallés, Spain). Upon arrival, the animals were housed in pairs for a week and then individually in animal quarters for another week to get them accustomed to oral administration. The rats had free access to food, standard chow (Teklad2014 from ENVIGO), containing (by energy) 20% protein, 13% fat, and 67% carbohydrates, and tap water. Room temperature was maintained at 22 °C with a 12 h light/12 h dark cycle (lights from 7:00 am to 7:00 pm). The animals were used in the experiments after this two-week acclimation period. The Animal Ethics Committee of University Rovira i Virgili (Tarragona, Spain) approved all procedures (CEA-OH/10715/3).

We tested three different protein sources: insect, beef, and almond. The design of the study involved the treatment of the ten animals in six different experimental days (Figure S1). In each experimental day two of the three protein sources were tested (5 animals per group). There was a washout period of a minimum of two days. Then, the rats were redistributed randomly in two other different groups to again test two of the three protein sources. After six experimental days, each rat was treated at least twice with each protein. We administered each protein source 20 times. The experimental design ensured that the animals received the same protein more than once. For this reason, for the calculations we considered the mean of each of the 10 animals (each one with 2–4 administrations of each protein source).

To test the protein sources each experimental day, animals were food deprived starting at 5:00 pm. We administered the protein load at 6:00 pm every treatment day. Rats were administered the different protein sources dissolved in the vehicle by controlled oral intake with a syringe. As vehicle, we used an artificial saliva with the same composition as described by Minekus et al. [33]. All animals received a dose of 300 mg protein/kg of body weight (BW). They had free access to food again when the dark cycle began at 7:00 pm. Food intake was measured 3, 12, and 20 h after the dark cycle began.

Control food intake of all the animals was measured twice during the washout periods.

### 2.7. Statistical Analysis

The results are expressed as the mean ± standard error of the mean (SEM). The sample size (*n*) for each variable is indicated in the corresponding figure description. A one-way ANOVA test and Tukey’s post-hoc test were used for multiple comparisons. *p*-values <  0.05 were considered statistically significant. These calculations were performed using XLSTAT 2020.1.2 software (Addinsoft, New York, NY, USA).

## 3. Results

### 3.1. Characterization of Digested Samples

**Figure 1:**
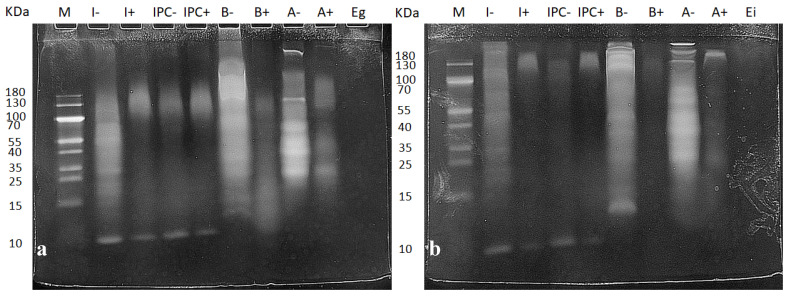
Protein hydrolysis of food after gastric (**a**) and intestinal (**b**) digestion. Samples marked with “+” are the digested ones and the samples marked with “−” are the negative controls for each digestion. The protein load was adjusted to 30 μg of protein in each lane, except for the enzyme controls, which were used without dilution. A molecular weight marker (10–180 kDa) was included. I, insect; IPC, insect powder concentrate; B, beef; A, almond; Eg, gastric enzyme control; Ei, intestinal enzyme control; M, molecular weight marker.

To ensure that the digestion protocol worked properly, we analyzed the protein hydrolysis with SDS-PAGE electrophoresis. As Figure 1 shows, the protein pattern between digested (+) and non-digested (−) samples was very different. The number and intensity of bands in the non-digested samples was much more pronounced than in the digested samples. The bands in the samples from the gastric phase (Figure 1a) were more intense for the digested samples than for the intestinal digested samples (Figure 1b). As the digestive process progressed the proteins became smaller, generating more peptides, and the bands become fainter. These small peptides are difficult to visualize in gel electrophoresis, and this is why the digested samples appear with less intensity in Figure 1b.

It is important to know a food’s composition in order to explain the effect that it can have at an intestinal level. We therefore examined the composition of both the gastrically and intestinally digested foods. Table 1 shows that after gastric digestion, the protein content was similar across our samples, as we defined. Since the digestion process itself diluted all the components to half at the beginning of each phase, after the intestinal phase, both protein and glucose levels were lower than they were in the gastric phase. As we did a centrifugation step to obtain homogeneous samples with which to treat the intestines, we obtained an upper lipidic phase and a precipitate with the debris and undigested remnants. This separation procedure had a clear effect on the triglyceride levels of the gastric digestions. They were very different from one another, with different proportions of triglycerides than in the original raw samples (Table S1). The triglyceride levels were higher after the intestinal phase, as expected due to the presence of bile salts.

### 3.2. Insect Gastric Digestion Stimulated Pig Duodenal Enteroendocrine Secretions Differently than Beef or Almond

**Figure 2:**
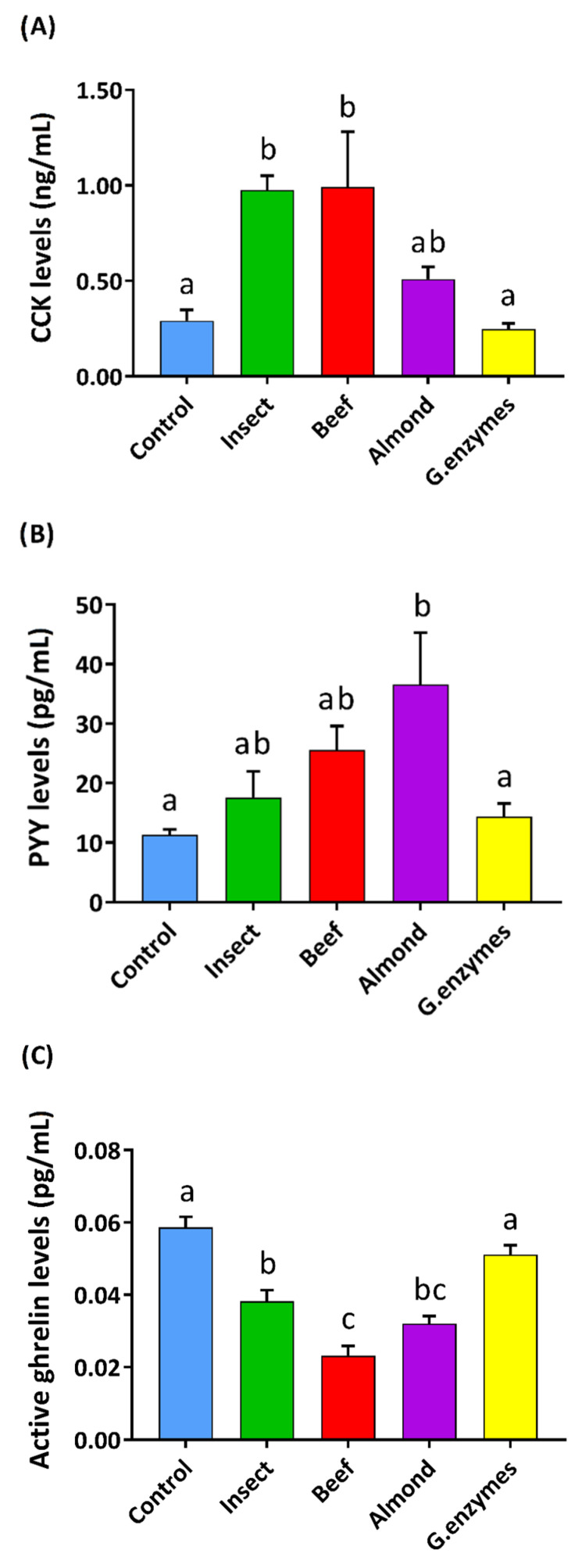
Effect of different gastric digestions (15 mg protein/mL) and the enzyme control on CCK (**A**), PYY (**B**), and active ghrelin (**C**) pig duodenum secretion in explants. The incubation period was 60 min for CCK and PYY and 90 min for active ghrelin secretion, all after 15 min of stabilization. The sample size was control (KRB buffer with D-glucose 10 mM) *n* = 9, treatments *n* = 6. Results are expressed as the mean ± SEM. One-way ANOVA and Tukey’s post-hoc test were used for multiple comparisons. Different letters (a, b, c) indicate significant differences (*p*-values < 0.05).

To evaluate the stimulation produced by these digested extracts on enterohormone secretion, we first tested the gastric in vitro digested samples on intestinal duodenum pig segments. Our results (Figure 2) showed that insect and beef digestions stimulated the secretion of CCK more than almond or the control. Additionally, insect and beef showed a similar effect on PYY secretion, but almond was the most effective at increasing PYY secretion. All of the foods tested significantly reduced ghrelin secretion, with beef being the most effective and insect the least. The enzyme control did not affect hormone release, as expected.

### 3.3. Insect Intestinal Digestion Induced GLP1 Secretion and Reduced Ghrelin Secretion in Human Colon Samples

**Figure 3:**
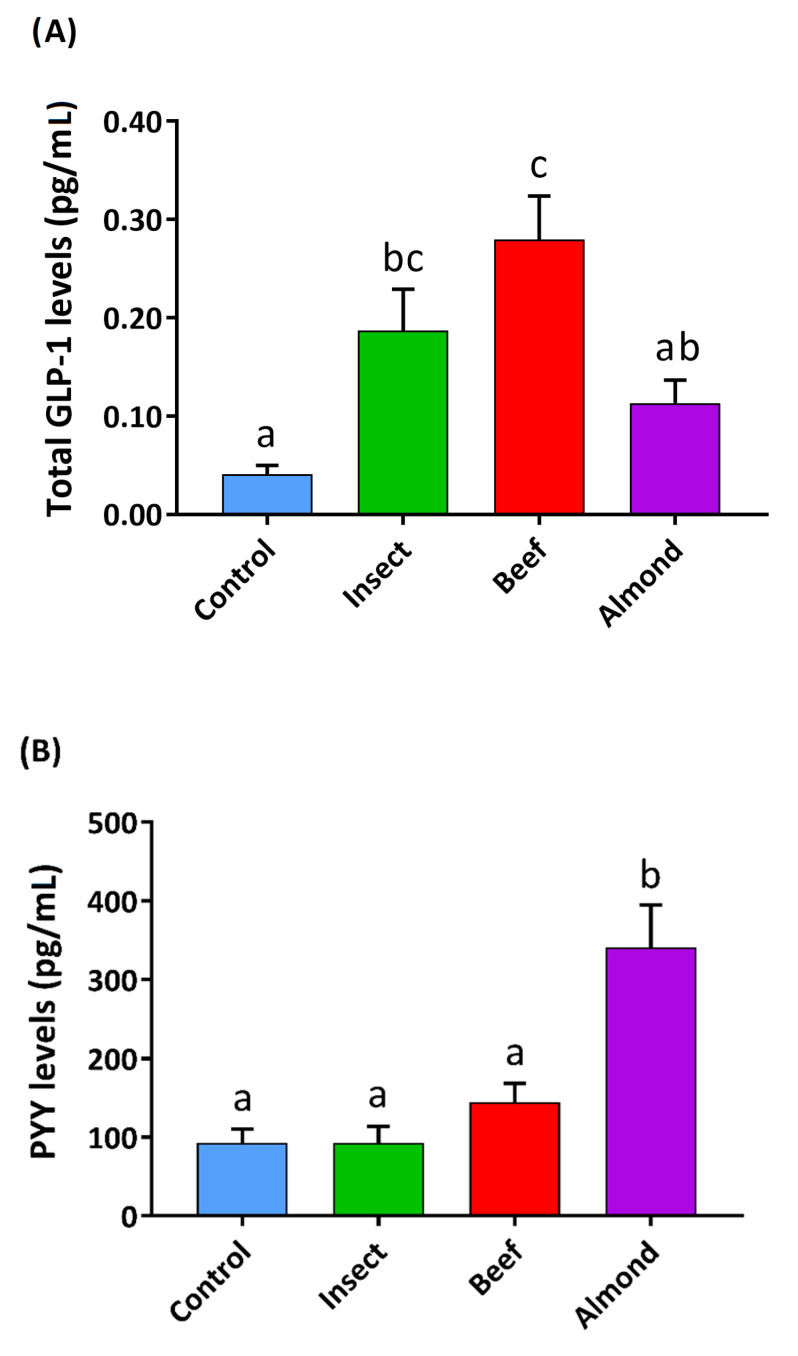

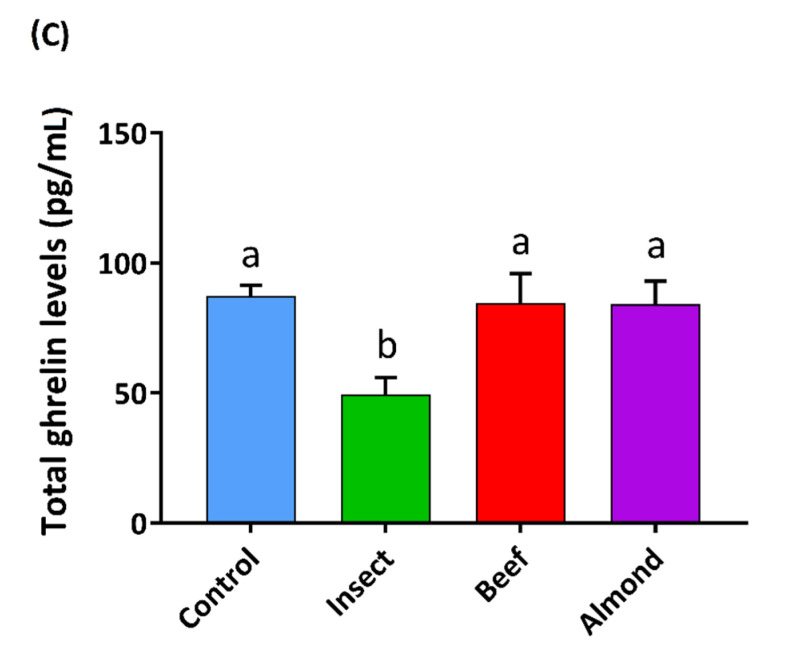
Effect of intestinal digestions of different foods (5 mg protein/mL) on total GLP-1 (**A**), PYY (**B**), and total ghrelin (**C**) human colon secretion in explants. The incubation period was 30 min after 15 min of stabilization. The sample size was *n* = 10 for control and treatment groups. Results are expressed as the mean ± SEM. One-way ANOVA and Tukey’s post-hoc test were used for multiple comparisons. Different letters (a,b,c) indicate significant differences (*p*-values < 0.05).

We were able to use human colon samples for the purpose of studying intestinal digestions. We also assayed *in-vitro*-digested IPC, as it is a commercially available product intended for human consumption. Because our focus was on the colon, and the amount of protein that reaches this site in vivo is expected to be lower, we used a smaller quantity of protein (5 mg protein/mL) with these samples. Figure 3 shows that raw insect and beef were the most effective in inducing the secretion of total GLP-1 (tGLP-1), as a measure of all GLP-1 secreted by these samples. IPC also increased tGLP-1 similarly to insect (0.19 ± 0.04 and 0.12 ± 0.02 pg/mL of GLP-1 for insect and IPC, respectively). PYY levels only increased after treatment with almond digestions. In the case of total ghrelin levels, the insect and IPC treatments (49.48 ± 3.41 and 43.35 ± 3.51 pg/mL, respectively) statistically limited ghrelin secretion compared to the control and to the other samples.

### 3.4. Buffalo Alphitobius diaperinus Powder Stimulated Food Intake in Rats

**Figure 4:**
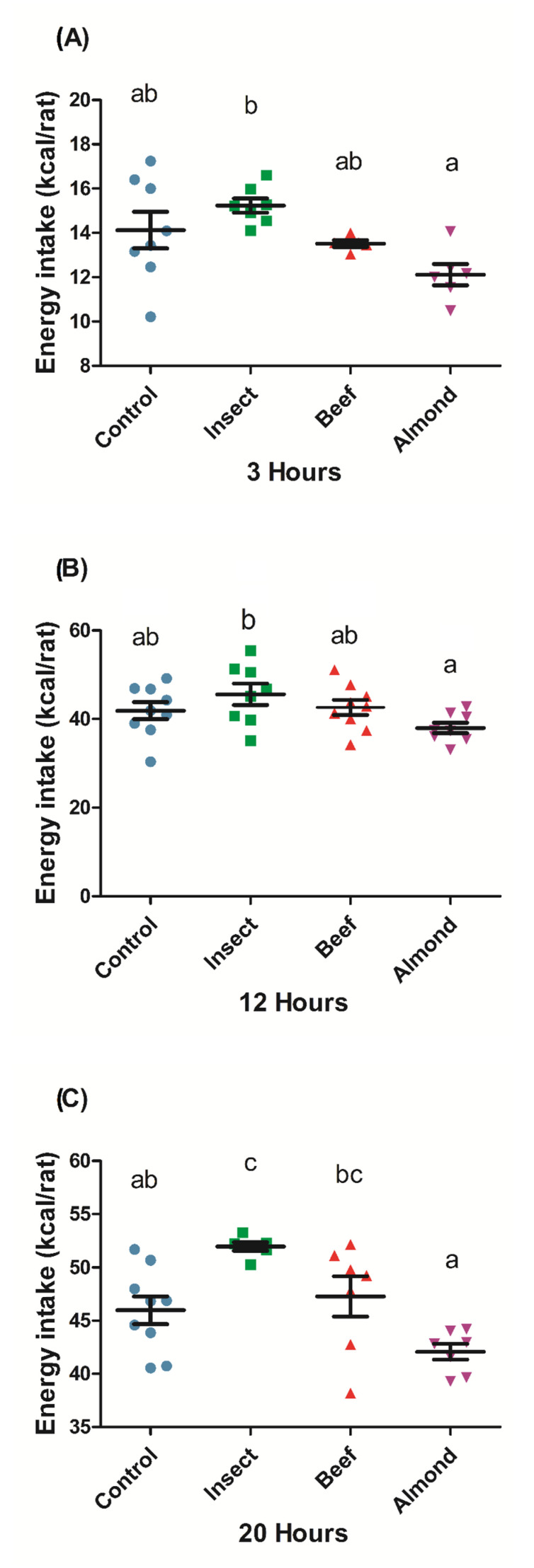
Energy intake after oral administration of a protein dose (300 mg/kg BW). Orally digested samples from insect, beef, or almond were administered to rats. Food intake was measured at three different intervals: 3 h (**A**), 12 h (**B**), and 20 h (**C**) after the beginning of the dark cycle. The control was an equivalent volume of tap water. The sample size was *n* = 10 rats per group. The results are expressed as the mean ± SEM. One-way ANOVA and Tukey’s post-hoc test were used for multiple comparisons. Different letters (a,b,c) indicate significant differences (*p*-values < 0.05).The circle, square, and triangle means feeding with different source of proteins.

As we found different secretions for the different assayed protein sources, we investigated their in vivo effects on food intake. We measured the energy intake in rats (Figure 4) after the oral administration of an equal protein dose of the three different foods (300 mg protein/kg BW, which corresponded to kJ (kcal) per rat: 2.7 (0.64) for insect, 2.0 (0.48) for beef, and 9.5 (2.27) for almond (composition detailed in Table S4). We observed that 3 h after the animals began eating (four hours after the treatment), the almond-treated rats ate less, while the insect-treated rats ate somewhat more compared to the control and beef groups. This effect was maintained twelve hours after the lights were switched off. In the final intake measure, 20 h after the start of the dark period, we observed that the almond-treatment group continued to consume less food than the other groups. The beef group showed a slight tendency towards higher intake than the control group, and the insect-treated group significantly increased its energy intake compared to the control group. These results suggest that after an administration of an equal dose of protein, insect protein stimulates food intake while almond protein provides the greatest satiating effect. The effect of insect and beef was also evident at 20 h if the energy content of the protein load (i.e., the total energy intake of each animal including the protein dose) is considered (Figure S2).

## 4. Discussion

In the present study, we compared the enterohormone secretion profile of three different *in-vitro*-digested protein sources, working on ex vivo systems of pig and human intestine. Our results show that the different protein sources produce a different secretome in both types of intestinal segments as well as differing effects on food intake responses in rats.

To test the effects of insect, beef, and almond on different intestinal segments (duodenum and colon), the food was first subjected to an in vitro digestion process, using a standardized methodology [32]. Our results for the SDS-PAGE electrophoresis are consistent with those of other authors [32]. Using beef samples [37], almond samples [38], or the same insect we used [39], they started with a similar pattern of proteins in the original sample and they disappeared similarly to ours after the different digestions. We can therefore assume that the protein in our samples was successfully hydrolyzed.

We assayed the duodenal enterohormone response to gastric digestion products working with pig samples. This species is considered a close and valid model for human studies [40], which facilitated the set-up for the procedure. Pig duodenum has been reported to produce high secretions of PYY, together with CCK secretions and measurable amounts of acylated ghrelin [41]. Our results showed that digested insect, almond, and beef significantly modulated the duodenal enterohormone profile, increasing the release of PYY and CCK and reducing that of ghrelin. In addition, the enterohormone secretion profile was different for each one, even though the treatments were performed with the same quantity of protein (15 mg/mL). This suggests that the total amount of protein is not the only factor responsible for the secretion of these satiety hormones. CCK secretion has been reported to be stimulated by lipids and protein [23,42], and PYY is secreted in proportion to caloric intake [42,43]. The reduction of ghrelin levels is proportional to the energy load and macronutrient content, and its suppression is greatly promoted by protein and less effectively by lipids [44,45]. Considering the composition of the digestion (Table S5), the largest component is protein, followed by triglycerides and glucose in a much smaller proportion. The differences in triglycerides also implies that the energy load of each treatment it is different: the insect is the most caloric, followed by almond and beef. Our results show that the increased secretion of PYY and CCK or the inhibition of ghrelin secretion did not correlate with the caloric content or triglycerides of the different food sources, suggesting that the quality of the protein is the primary factor responsible for the observed differences.

As observed with the duodenum, neither the energy load nor the macronutrient composition of the intestinally digested food (Table S6) explains the effects on enterohormone secretion in the colon. We also found differences among foods in PYY and ghrelin secretions, as well as in tGLP-1 secreted in this intestinal segment. We worked with complex mixtures of dietary components that underwent a transformation during the digestion process. We simulated the in vivo digestion process with the INFOGEST harmonized protocol [32], which is very close to in vivo pig protein hydrolysis, as demonstrated by Egger et al. [46]. It has been shown that protein hydrolysates have different effects on the enteroendocrine system, mainly due to the presence of specific peptides [47]. The amino acids and di-, tri-, and oligopeptides generated during protein digestion interact with EECs [25]. These resulting peptides differ depending on the source of the protein, the enzymes present, and the physicochemical conditions of the process. Here we include a new protein which has not been previously assayed on enterohormone secretions. Each intestinal segment responds to nutrient intake differently. In addition, the compositions of gastric and intestinal digestion are different, and this is likely true regarding the resulting peptides as well. Further characterization of the hydrolysis products of our different samples will help to elucidate the primary factor responsible for each enterohormone secretion.

Differences between the secretomes could give rise to differing physiological effects, such as changes in food intake patterns. Protein has been reported to have a good satiety capacity compared to other macronutrients [29,48]. In this study we show that beef, almond, and insect produced different effects on food intake after the administration of an equivalent amount of protein. We found that the orally digested almond was the most satiating in rats. This could be due to the fact that the caloric load administered for almond was higher than that for the other foods (Table S4). The total energy intake, including the caloric load of the treatments, did not differ from the control (Figure S2). The satiating effect of almond disappeared in the overall energy intake, which was similar to that observed in other experiments where, although it had the highest satiety quotient, almond total energy intake did not differ from the other treatments [48]. We did not anticipate finding that administering insect protein would increase food intake, even when considering the total energy intake. These differential effects cannot be explained by the secretome found in the ex vivo studies, since, overall, we observed an increase in anorexigenic hormones and a reduction in orexigenic ghrelin in proportions that did not correlate with the effects on the intake of each food. Actually, in vivo hormonal profile is complex and depends not only on the direct effects of food stimulation of receptors, but also on the indirect paracrine effects of secreted hormones, the inactivation of these hormones, and other factors. In this paper, we did not aim to fully decipher the mechanism of action of each protein source, but we show that they acted differently on the secretome and that they had different effects on food intake. Differences in food intake after the administration of an equivalent protein dose have previously been reported in the literature. In a study in rats, a high-protein meal composed of egg white suppressed food intake while a wheat-gluten-based meal containing the same percentage of protein, fat, and carbohydrates increased food intake [49]. Other examples are found in the literature for human studies. A whey pre-meal was found to similarly reduce food intake to the same protein load of casein [50], soy, turkey, egg, or tuna [51,52]. In the digestion process, as previously discussed, proteins are hydrolyzed in different ways according to their physicochemical properties. A clear example is the difference between whey protein, which enters the jejunum mostly intact, and casein, which appears mainly in the form of degraded peptides, attributed to the precipitation of casein in the acid media of the stomach and the longer exposure to gastric hydrolysis [53]. Therefore, another important factor that may explain these differences in food intake regulation could be the digestibility of each food. It can limit the derived products of hydrolysis (i.e., peptides and amino acids), which can modify the secretome obtained in each case. Of the indices that evaluate protein quality in relation to digestibility, one of the most widely adopted is the protein digestibility corrected amino acid score (PDCAAS) [54]. Raw almond has been classified with a PDCAAS value of around 45 [55] and beef with a value of 92 [56], approaching the maximum of 100. Little is known in this regard for insect species, and each species has very different particularities. The chitin present in insects has been shown to affect protein digestibility [57]. In a study with the insect species *Tenebrio molitor*, the protein digestibility was assessed in different fractions, showing that the supernatant fraction had higher protein digestibility compared to the whole-insect or the pellet fractions [58].

## 5. Conclusions

In conclusion, in this study we demonstrated that insect, beef, and almond modulate food intake differently when administered in equal amounts of protein: *Alphitobius diaperinus* increased food intake in rats, while almond limited it. We also showed that the digestion of these food sources modulates enterohormone release. The analysis of the amino acid and peptide composition of gastric and intestinal in vitro digestion could lead to a better understanding of the enterohormone secretion profile we obtained with these ex vivo experiments. The quantity, source, bioavailability, and hydrolysis of proteins are key points that affect their interaction with the EECs, in both ex vivo and in vivo conditions.

## Figures and Tables

**Table 1:** Protein, glucose, and triglyceride composition of digested food samples after gastric and intestinal in vitro digestion.

Samples		Protein mg/mL	Triglycerides mg/mL	Glucose mg/mL
Gastric digestion	Insect	26.60	5.84	0.20
	IPC	31.49	15.30	0.22
	Beef	21.60	0.92	0.09
	Almond	26.55	2.24	0.12
	Enzymes	0.65	0.05	n.d.
Intestinal digestion	Insect	15.02	8.97	0.09
	IPC	14.38	12.91	0.08
	Beef	9.28	0.70	0.06
	Almond	10.29	9.44	0.1
	Enzymes	0.62	0.05	n.d.

n.d.: not determined.

## Supplementary Materials

The following are available online at https://www.mdpi.com/2072-6643/12/8/2366/s1, Figure S1: Rat intake experimental design, Figure S2: Total energy intake after an oral administration of a protein dose (300 mg/kg BW) from insect, beef, or almond in rats at different time points, Table S1: Nutritional composition of raw insect, IPC, almond, and beef, Table S2. Web addresses where the protocols of the ELISA kits used to obtain the results shown in this article can be obtained, Table S3. Descriptive statistics and biochemical parameters of the colon donor patients, Table S4. Nutritional composition (according to data provided by producers) of protein treatments administered to rats. Data presented are adjusted to rat body weight (250 g on average). Protein dose was equal for all treatments, Table S5. Nutritional composition of gastrically digested samples used to treat pig duodenum explants. Protein content was adjusted to 15 mg/mL, Table S6. Nutritional composition of intestinally digested samples used to treat human colon explants. Protein content was adjusted to 5 mg/mL.
