# Supplementary material for: Gastrointestinally Digested Protein from the Insect Alphitobius diaperinus Stimulates a Different Intestinal Secretome than Beef or Almond, Producing a Differential Response in Food Intake in Rats
Source: Nutrients. 2020 Aug 7;12(8):2366. doi: 10.3390/nu12082366 (PMC7468914; doi:10.3390/nu12082366)
Supplement: Supplementary file 1 [file nutrients-12-02366-s001.pdf]

Table S1. Nutritional composition of raw insect, IPC, almond, and beef.

Composition	Insect	IPC	Beef	Almond
Energy (kJ)	2131.80	1813.70	547.58	2524.72
Protein (g)	59.60	58.30	20.70	20.00
Total lipids (g)	28.70	22.30	5.40	53.50
Carbohydrates (g)	2.70	4.40	0.00	3.50
Fiber (g)	3.70	4.20	0.00	14.30

The data were provided by the manufacturers—except for beef, the data for which were obtained from Moreiras et al., 2013 [31].

Table S2. Web addresses where the protocols of the ELISA kits used to obtain the results shown in this article can be obtained.

Hormone	Intra-Assay Variation	Web Address (*Consulted 27 July 2020*)
Pig samples		
Active Ghrelin	< 5%	https://www.merckmillipore.com/ES/es/product/Rat-Mouse-Ghrelin-active-ELISA,MM_NF-EZRGRA-
CCK	< 10%	https://www.phoenixpeptide.com/products/view/Assay-Kits/EKE-069-04
PYY	< 10%	https://www.phoenixpeptide.com/products/view/Assay-Kits/FEK-059-03
Human samples		
Total Ghrelin	< 2%	https://www.merckmillipore.com/ES/es/product/Human-Ghrelin-total-ELISA,MM_NF-EZGRT-
Total GLP1	< 2%	https://www.merckmillipore.com/ES/es/product/Multi-Species-GLP-1-Total-ELISA-EZGLP1T-36K,MM_NF-EZGLP1T-
PYY	< 10%	https://www.phoenixpeptide.com/products/view/Assay-Kits/FEK-059-02

Table S3. Descriptive statistics and biochemical parameters of the colon donor patients.

Characteristics	All (*n*= 10)	Proximal Colon Donors (*n* = 5)	Distal Colon Donors (*n* = 5)
Age (years) *n* = 9			
> 60	9 (100%)	5 (100%)	4(100%)
Gender *n* = 10			
Male	8 (80%)	3 (60%)	5 (100%)
Female	2 (20%)	2 (40%)	0 (0%)
Tobacco consumption (*n* = 8)			
No	6 (75%)	4 (80%)	2 (66.7%)
Yes	2 (25%)	1 (20%)	1 (33.3%)
Alcohol consumption (*n* = 6)			
Never	1 (16.67%)	0 (0 %)	1 (33.3%)
< 30 g/day	3 (50%)	1 (33.3%)	2 (66.7%)
> 30 g/day	2 (33.3%)	2 (66.7%)	0 (0%)
		BMI (*n* = 10)	
(kg m^{-2})	26.79 ± 1.08	26.57 ± 1.0	27.01 ± 2.05
Glucose (*n* = 10)			
(mM)	6.34 ± 0.55	5.71 ± 0.59	6.98 ± 0.91
Total cholesterol (*n* = 9)			
(mg dL^{-1})	177.67 ± 7.03	179.8 ± 8.62	176.0 ± 11.48
Triglycerides (*n* = 7)			

(mg dL^{-1})	121.43 ± 12.2	110 ± 29.26	130 ± 5.96

Data are presented as number of patients (percentage) or mean ± SEM.

Table S4. Nutritional composition (according to data provided by producers) of protein treatments administered to rats. Data presented are adjusted to rat body weight (250 g on average). Protein dose was equal for all treatments.

**Composition**	**Treatment Composition (per rat)**		
	**Insect**	**Beef**	**Almond**
Energy (kJ)	2.70	2.00	9.50
Protein (mg)	75.00	75.00	75.00
Total lipids (mg)	36.10	19.60	200.60
Carbohydrates (mg)	3.40	0.00	13.10
Fiber (mg)	4.70	0.00	53.60

Table S5. Nutritional composition of gastrically digested samples used to treat pig duodenum explants. Protein content was adjusted to 15 mg/mL.

**Sample**	**Protein**	**Triglycerides**	**Glucose**
	mg/mL	mg/mL	mg/mL
Insect	15.00	3.29	0.11
Beef	15.00	0.64	0.06
Almond	15.00	1.27	0.07

Table S6. Nutritional composition of intestinally digested samples used to treat human colon explants. Protein content was adjusted to 5 mg/mL.

**Sample**	**Protein**	**Triglycerides**	**Glucose**
	mg/mL	mg/mL	mg/mL
Insect	5.00	2.99	0.03
Beef	5.00	0.38	0.03
Almond	5.00	4.59	0.05
IPC	5.00	4.49	0.03

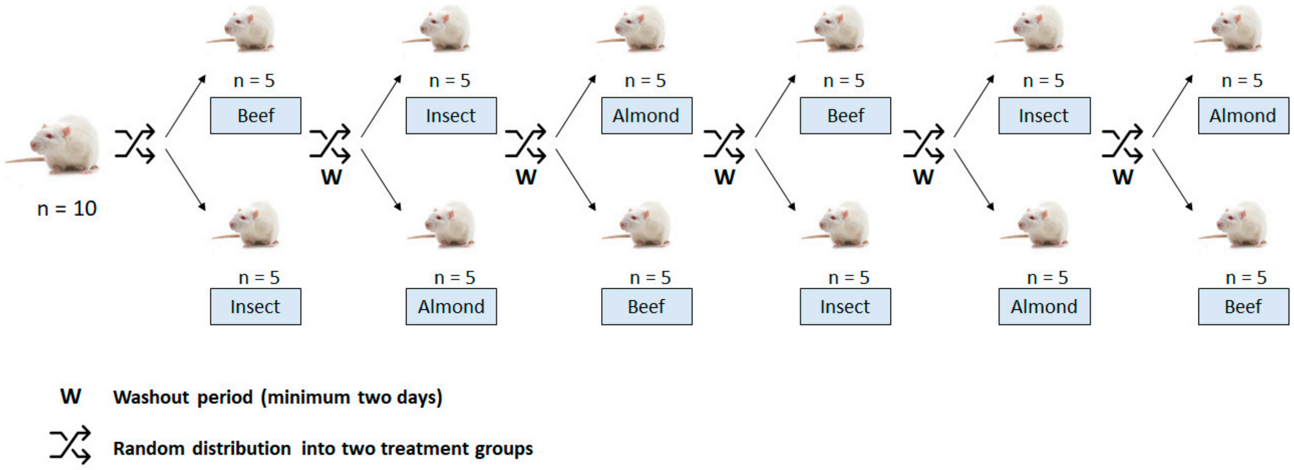

Figure S1. Rat intake experimental design. Three different protein sources were tested in six experimental days. In each of these days, two of the three protein sources were tested (5 animals per

group). After each administration, the rats were redistributed randomly into new treatment groups. There was a washout period of a minimum of two days.

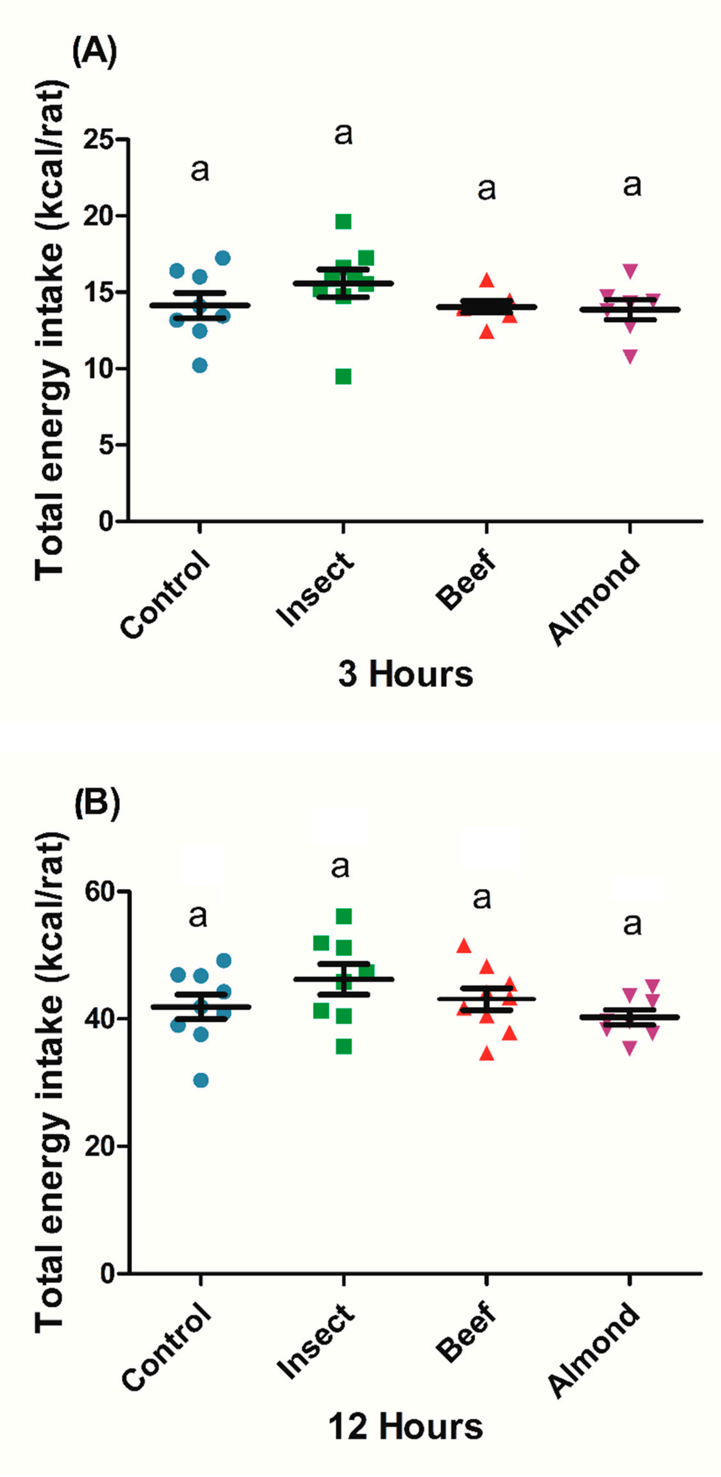

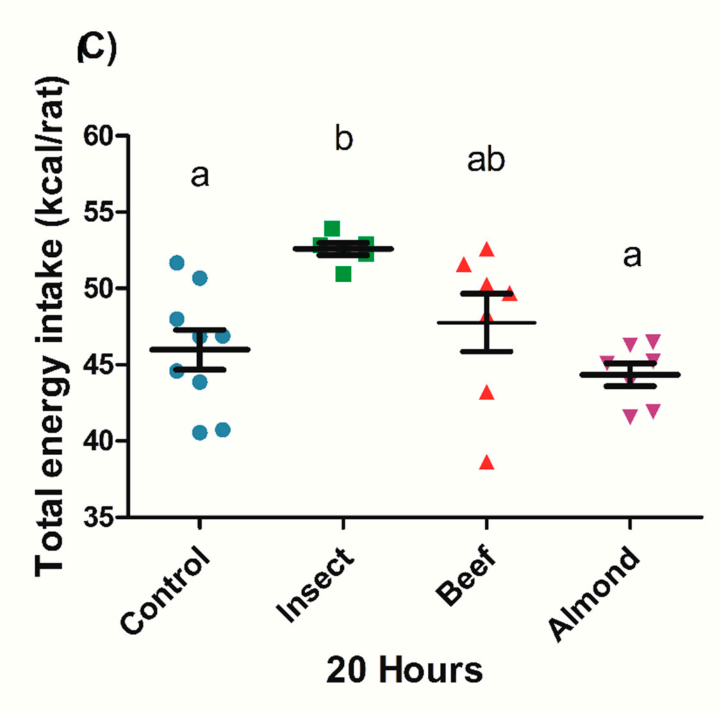

Figure S2. Total energy intake after an oral administration of a protein dose (300 mg/kg BW) from insect, beef, or almond in rats at different time points: 3 h (**A**), 12 h (**B**), and 20 h (**C**) after the dark cycle began. The caloric load of the treatments is included in the total energy intake of each group. The control was an equivalent volume of tap water. The sample size was $n = 10$ per group. The results are expressed as the mean ± SEM. One-way ANOVA was used for multiple comparisons. Different letters (a,b,c) indicate significant differences (p-values < 0.05).
